# Analysis of the Strength of Polyamide Used for High Pressure Transmission of Hydrogen on the Example of Reinforced Plastic Hoses

**DOI:** 10.3390/ma18071402

**Published:** 2025-03-21

**Authors:** Natalia Dawicka, Beata Kurc, Xymena Gross, Jakub Tomasz, Katarzyna Siwińska-Ciesielczyk, Agnieszka Kołodziejczak-Radzimska

**Affiliations:** 1 Institute of Chemistry and Electrochemistry, Faculty of Chemical Technology, Poznan University of Technology, Berdychowo 4, PL-60965 Poznan, Poland; n.dawicka4@gmail.com (N.D.); xymena.gorka@student.put.poznan.pl (X.G.); jakub.tomasz@student.put.poznan.pl (J.T.); 2 Institute of Chemical Technology and Engineering, Faculty of Chemical Technology, Poznan University of Technology, Berdychowo 4, PL-60965 Poznan, Poland; katarzyna.siwinska-ciesielczyk@put.poznan.pl (K.S.-C.); agnieszka.kolodziejczak-radzimska@put.poznan.pl (A.K.-R.)

**Keywords:** hydrogen, materials, hydrogen storage, hydrogen transfer, mechanical properties, high pressure conditions, hydrogen exposure

## Abstract

The purpose of this study is to evaluate the strength of polyamide utilized in high pressure hydrogen transmission, exemplified by reinforced plastic hoses. The research encompasses a comprehensive investigation of materials employed in hydrogen infrastructure, focusing on their barrier and mechanical properties. It addresses challenges associated with hydrogen storage and transport, presenting various types of tanks and hoses commonly used in the industry and detailing the materials used in their construction, such as metals and polymers. Two materials were analyzed in the study; one new material and one material exposed to hydrogen. Key mechanisms and factors affecting gas permeation in materials are discussed, including an analysis of parameters such as fractional free volume (FFV), solubility coefficient (S), diffusion coefficient, and permeability coefficient. Methods for evaluating material permeation were outlined, as they are essential for assessing suitability in hydrogen infrastructure. Experimental analyses included Fourier Transform Infrared Spectroscopy (ATR), differential thermal analysis (DTA), scanning electron microscopy (SEM), and Energy dispersive X-ray spectroscopy (EDS). These techniques provided detailed insights into the structure and properties of polyamide, allowing for an assessment of its performance under high pressure hydrogen conditions. Pressure was identified as a critical factor influencing both the material’s mechanical strength and its hydrogen transport capability, as it affects the quantity of adsorbed particles. According to the DTA investigation, the polyamide demonstrates minimal mass loss at lower temperatures, indicating a low risk of material degradation. However, its performance declines significantly at higher temperatures (above 350 °C). Up to 250 °C, the material shows no notable decomposition occurred, suggesting its suitability for certain applications. The presence of functional groups was found to play a significant role in gas permeation, highlighting the importance of detailed physicochemical analysis. XRD studies revealed that hydrogen exposure did not significantly alter the internal structure of polyamide. These findings suggest that the structure of polyamide is well-suited for operation under specific conditions, making it a promising candidate for use in hydrogen infrastructure. However, the study also highlights areas where further research and optimization are needed. Overall, this work provides valuable insights into the properties of polyamide and its potential applications in hydrogen systems.

## 1. Introduction

Due to its small particle size and high diffusivity, hydrogen gas can easily permeate polymeric materials. To enhance their hydrogen barrier properties, functional groups can be incorporated into polymer chains. The introduction of polar groups, such as hydroxyl, carboxyl, and nitrile groups, improves intermolecular interactions, reducing free volume and creating a denser polymer matrix, which decreases hydrogen permeability [1,2,3]. The polymer’s crystallinity can be increased by the addition of chlorine groups, which leads to the formation of a more ordered structure that impedes hydrogen diffusion. The incorporation of aromatic rings and bulky substituents can increase the polymer density, limiting the available pathways for hydrogen permeation. Furthermore, functional groups capable of forming hydrogen bonds, such as hydroxyl, amino, and carboxyl groups, create strong intermolecular networks that further reduce permeability.

Hydrogen storage methods are a key area of research in the context of the development of hydrogen technologies [4]. There are several main methods of storing this gas, each of which has its advantages and limitations. One of these is the storage of hydrogen in compressed form, where the gas is stored under high pressure in cylinders. Another method is to cool hydrogen to very low temperatures, turning it into liquid hydrogen (LH_2_). It is also possible to absorb hydrogen by porous materials, such as metals [5,6]. Each of these methods has its applications in various areas, from transport to energy storage, and research on them aims to discover optimal solutions in terms of efficiency, safety, and economy.

Unlike hydrogen tanks, there are no standardized requirements for the construction of hoses for transmitting this element. Similar to tanks, the oldest material used to transmit hydrogen was carbon steel. Numerous sources indicate that no problems with hydrogen embrittlement were observed in low-pressure applications [7,8]. Due to the need to increase the efficiency of transporting this medium, compressing the gas to obtain a larger gravimetric capacity necessitated compression. For example, at a pressure of 345 bar, we obtain a density of 23 kg/m^3^, while at atmospheric pressure the bulk density is only 0.09 kg/m^3^. Due to the increase in the mass of hydrogen per unit volume, the carbon steels previously used no longer allow for the safe transport of this element [8]. Hoses with thermoplastic linings have a similar structure to type IV tanks.

**Figure 1:**
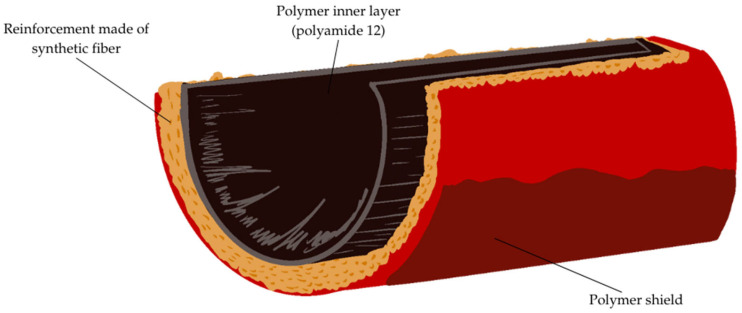
Diagram of the construction of a flexible hose for hydrogen transfer [8].

Research and development in the field of composite hoses for transmitting hydrogen is not very advanced, but there are many proven technologies for producing hoses for transmitting compressed natural gas (CNG) that can be a reference point. Hoses for high pressure gas transmission have a multi-layer structure: a polymer inner layer, reinforcement (polyester or aramid), and an outer cover that prevents mechanical damage. An example of the construction of a layered hose is shown in Figure 1.

During the life of thermoplastic hoses, there are repeated cycles during which hydrogen comes into direct contact with the material. This is accompanied by cyclical temperature changes in the range of −40 to 85 °C, cyclical pressure changes in the extreme range of 1 bar~350/700 bar (depending on the application), and variable humidity values.

Hydrogen atoms, regardless of their source, can diffuse into steels and alloys, causing hydrogen damage at ambient or low temperatures through a number of different mechanisms: hydrogen bubbles, hydrogen embrittlement, and soft zone cracking. At high temperatures, hydrogen atoms can react with carbon in stainless steel and alloys, causing surface decarburization, which also contributes to structural weakness and possible damage [9,10,11]. Due to numerous problems associated with the use of steel in hydrogen infrastructure, polymeric materials have been used in hydrogen transmission applications. As in the case of type IV tanks, currently used hydrogen hoses have a polymer inner layer. Due to their properties, polyamides [7,11,12] and HDPE show promising results in terms of low hydrogen penetration through the walls. Polyamide, also known as nylon, is a condensation polymer whose basic structure consists of repeating amide units (–CONH–). A typical polyamide is nylon-6,6, which is made from hexamethylenediamine and adipic acid [13]. Polyethylene, on the other hand, is an addition polymer composed of repeating ethylene units (–CH_2_–CH_2_–). A comparison of the permeation properties is provided in Supplementary Materials.

Various storage methods, each with unique benefits and limitations, are being developed and refined to serve different applications, from transportation to large-scale energy storage. One primary approach to hydrogen storage is compression, where hydrogen is stored in high pressure tanks, typically between 350 and 700 bar. This method is straightforward and enables rapid refueling, making it well-suited for transportation applications such as fuel cell vehicles. However, high pressure storage demands robust materials to withstand the pressure, raising safety concerns about potential leaks and tank integrity under extreme conditions. Another approach is liquid hydrogen (LH_2_) storage, which involves liquefying hydrogen at cryogenic temperatures, approximately −253 °C. Liquefied hydrogen is much denser than gaseous hydrogen, allowing for more energy storage within a smaller volume, which is advantageous for long-distance transportation. Nevertheless, this method faces challenges due to the energy-intensive liquefaction process and the need for insulated storage tanks to maintain the low temperatures, making it complex and costly.

Solid-state storage represents a third method, in which hydrogen is absorbed or adsorbed in solid materials such as metal hydrides or metal-organic frameworks (MOFs). This approach offers higher volumetric density and improved safety due to the lower pressures involved compared to gaseous storage. However, the release of hydrogen in this form can be slow, as it often requires heating to effectively release the stored hydrogen.

Polymers are integral to the design of modern hydrogen storage systems, particularly in high pressure applications. Type IV hydrogen storage tanks, commonly employed in vehicles and other mobile applications, utilize polymer-based liners—typically made from high density polyethylene (HDPE) or polyamide—that are reinforced with carbon fiber. These polymer liners serve two main functions: they act as gas barriers to minimize hydrogen permeation, addressing one of the primary challenges in hydrogen storage, and they maintain structural integrity under high pressure to ensure the system’s safety and reliability. Despite their advantages in terms of weight and flexibility, polymers face challenges with gas permeation; hydrogen molecules can diffuse through polymer matrices, potentially leading to a gradual loss of stored hydrogen over time, which is critical to both the efficiency and safety of storage systems [13,14,15].

The comparison between polymer liners and metal liners in hydrogen storage highlights differences in permeability and structural characteristics (Table 1). Polymers generally have higher hydrogen permeability than metals, allowing hydrogen molecules to diffuse through them more easily. Although polymers can be engineered for improved barrier properties—such as by increasing liner thickness or adding permeation-resistant additives—they remain susceptible to some degree of permeation due to their molecular structure. Commonly used polymers like polyamide and HDPE may require additional measures to minimize hydrogen loss. While metals are better at preventing hydrogen loss, some metals may corrode in hydrogen environments, affecting long-term performance, although coatings or alloying can improve corrosion resistance.

In summary, while polymer liners offer advantages in terms of weight and flexibility, they face challenges related to hydrogen permeation. Metal liners provide superior gas barrier properties but come with trade-offs in weight and cost. The choice between polymer and metal liners will depend on the specific application requirements, including safety, efficiency, and operational conditions. Our research revealed the advantages of polyamide hoses used in the hydrogen transfer over the metal materials. In addition, these results serve as a basis for further exploration in this field.

The gas permeability of polymers is influenced by the molecular structure of the polymer, including the presence and nature of functional groups. Functional groups can significantly change the physical properties of the polymer, including density, crystallinity, and intermolecular interactions, thereby affecting its gas barrier properties [1]. Various functional groups influence gas permeability, with particular emphasis on hydrogen, which poses unique challenges due to its small molecular size. Hydrogen flow can lead to the mechanical removal of fine inorganic particles or material degradation products, especially if high gas flow rates are present. Elastomers and polymers used in hydrogen transport hoses contain stabilizing additives. Prolonged contact with hydrogen can lead to their degradation or leaching, which alters the inorganic content of the material [1]. Several key functional groups known to reduce gas permeation in polymers is discussed in Supplementary Materials (Table S1).

In response to the growing demands for safety and environmental protection, a new flame retardancy method has been developed that eliminates the need for traditional flame retardants, which often contain halogens or other toxic elements that can pose health and environmental hazards. A new flame retardant method eliminates traditional halogen-based flame retardants, improving safety and environmental protection. In this study, an aliphatic polyamide is analyzed using hydrogen bonding at ambient temperatures and structural rearrangement at high temperatures. Its phenylimido units with hydroxyl groups improve fire resistance and mechanical properties. Consisting of carbon, hydrogen, oxygen, and nitrogen, the copolymer exhibits stability under thermal stress. More information in Table S1.

This study investigates the effects of hydrogen exposure on the properties of polyamide, a material critical for hydrogen storage applications. Two samples were analyzed: one before and one after 10 h of hydrogen exposure, allowing for the assessment of structural and property changes under these conditions. While the focus is on a single material and a limited number of samples, this research provides a crucial preliminary step in understanding the mechanisms of polymer degradation caused by hydrogen exposure. Material degradation in such applications is a significant concern, yet the body of scientific literature on this subject is limited. Therefore, the findings of this study offer a valuable foundation for future research, including comparative studies of other polymers and composites, to identify optimal materials for hydrogen storage technologies.

## 2. Experimental

### 2.1. Materials

The hydrogen experiment was conducted with due care, in accordance with the procedures for working with flammable gases. Hydrogen (from AirProducts company based in Warsow, Poland) was supplied from a small, handy 10-L cylinder through the analyzed hydrogen-resistant hose. The gas pressure was 1 bar, the flow was controlled at 50 mL/min, and the exposure temperature was 25 °C (laboratory conditions). The experiment lasted 10 h, and the hydrogen used had a purity of 99.999%. The entire process took place in a well-ventilated room, and the system’s tightness was regularly checked to minimize the risk of leakage. The hose manufacturer, Tubes International, is based in Poznan, Poland.

### 2.2. Experiment and Characterization

#### 2.2.1. Thermogravimetric Analysis (TGA/DTA/DTG) 

Thermogravimetric Analysis (TGA/DTA/DTG) is a technique that measures changes in sample mass as a function of temperature. It is used to study the thermal stability of materials and their degradation mechanisms. In the context of the inner layer of a plastic hose made of polyamide, TGA allows for the assessment of how the chemical composition and additives affect the material’s barrier properties against hydrogen transmission [16].

TGA testing of polyamide was carried out according to the following stages:

- Sample preparation: a fragment of the inner layer of the plastic hose (polyamide) was cut out into a square shape with dimensions of 0.5 cm × 0.5 cm, ensuring the appropriate size and weight of the sample. A new hose sample (1) and a sample that had been in contact with hydrogen gas for 10 h (2) were used for the study. Sample 2 was part of the system: hydrogen cylinder—fuel cell.
- The material was dried to remove any moisture that could affect measurement accuracy. The thermogravimetric analyzer TG 209 F3 Tarsus^®^ (Netzsch, Selb, Germany) was used to perform the analysis. The sample was placed in the analyzer oven. The temperature range was set from 30 °C to 1060 °C with a gradual temperature increase of 10 K/min in an inert gas atmosphere (nitrogen).

The thermal stability of the tube samples was assessed using thermogravimetric analysis (TG) and differential thermal analysis (DTA). A TG analyzer Jupiter STA 449 F3 from Netzsch, Selb, Germany, was used to measure the mass loss of the samples over a temperature range from 20 to 1000 °C at a heating rate of 10 °C/min under a nitrogen flow of 10 mL/min. 5 mg of sample material was used for each test. Alumina, which remains stable under the experimental conditions, was used as a reference. TG analysis monitors the mass changes of the sample when it is subjected to controlled heating, displaying these changes in the form of a thermogram. DTA, on the other hand, detects thermal effects related to physical or chemical changes occurring in the sample during the heating process [17].

#### 2.2.2. Differential Scanning Calorimetry (DSC) Measurements

The samples were first melted and then quenched, preparing them for measurement. Differential Scanning Calorimetry (DSC) measurements were conducted using the NEXTA DSC600 High Sensitivity Differential Scanning Calorimeter (Mettler-Toledo, Warszawa, Poland). Each measurement was performed on a 10 mg sample over a temperature range of 0 to 320 °C, with a heating rate of 10 °C min^−1^.

#### 2.2.3. Attenuated Total Reflectance Infrared Spectroscopy (ATR-IR)

ATR-IR analysis was conducted to examine the chemical structure of the polyamide inner layer. Following the separation of the inner layer from the hose braid and outer layer, samples were prepared to ensure good contact with the ATR crystal surface for accurate results. Infrared radiation was directed onto the ATR crystal, with total internal reflection occurring at the sample crystal interface, generating an ATR spectrum. Two polyamide samples were analyzed before exposure to hydrogen, and two samples were analyzed after exposure to hydrogen at low pressure (up to 2 bar, 20 °C) The amounts of compounds used to produce the pellet were 200 mg KBr and approximately 1.5 mg of sample. Both substances were mixed together and additionally ground in a mortar. The mixture was pressed under pressure on a PIKE Technologies hydraulic press. The pellet was analyzed in a Vertex 70 apparatus manufactured by Bruker (Billerica, MA, USA) [16].

#### 2.2.4. X-Ray Diffraction (XRD)

XRD analysis was performed on the pure polyamide (sample 1) and the polyamide exposed to hydrogen (sample 2) to investigate their crystal structures. Measurements were performed using a Bruker AXS D8 Advance diffractometer configured in Bragg-Brentano geometry (Bruker, Billerica, MA, USA). The system was equipped with a VANTEC-1 detector and a Ni filter to minimize fluorescence, using Cu Kα radiation (λ = 154 pm). Diffraction patterns were collected over a 2θ range from 10° to 90°, with a step size of 0.014° and an acquisition time of 1 s per step to provide high resolution measurements. The choice of the Bragg-Brentano geometry was ideal for obtaining well-defined peak intensities and detecting subtle changes in crystallinity, while the wide scanning range facilitated the observation of potential secondary phases or structural rearrangements. These settings enabled comprehensive evaluation of both primary and secondary crystalline features, allowing detailed comparison of neat and hydrogen-treated polyamide samples.

#### 2.2.5. Scanning Electron Microscopy (SEM) and Energy Dispersive X-Ray Spectroscopy (EDS)

The surface morphology, shape, and size of the area were analyzed by scanning electron microscopy (SEM) with an EVO40 microscope from Zeiss AG, Oberkochen, Germany. To obtain high resolution imaging, the material must be coated with a conductive layer such as platinum or gold to prevent the accumulation of excess surface charge on the sample. The tubes were cut to observe the inner part, which had direct contact with the gas. To obtain high resolution imaging, the material must be coated with a conductive layer, such as platinum or gold to prevent excess surface charge accumulation on the sample.

Energy dispersive X-ray spectrometry (EDS) is an analytical technique used to investigate the chemical composition of materials. Since the application of a conductive coating affects the EDS analysis by introducing strong peaks from the coating material, SEM imaging and EDS analysis were performed on separate samples. For SEM imaging, the samples were coated with a conductive layer, while uncoated samples were used for EDS analysis. This approach provides an accurate assessment of the elemental composition of the inner polyamide layer, which is crucial for assessing its barrier properties in terms of hydrogen transfer.

#### 2.2.6. Fractional Free Volume (FFV)

Gas permeability measurements are an indirect yet widely used method for estimating fractional free volume (FFV) in polymers. Because FFV represents the unoccupied space in the polymer matrix, it directly affects how gases diffuse and permeate through the material. Higher FFV values generally correlate with increased gas permeability due to the larger void spaces available for molecular transport. Uncertainty in FFV values results from variations in repeated measurements that were performed to assess the repeatability and reliability of the obtained values. The standard deviation of these repeated measurements was used to estimate the uncertainty. Computational models assist in predicting FFV by simulating polymer chain packing and free volume distribution. Model calculations for FFV are based on theoretical or empirical equations, such as polymer density, predicted van der Waals volume, or other variables that are functions of temperature. An important step is to account for errors resulting from measurements, such as density, and uncertainties in the model. Errors can be estimated from repeated measurements or errors resulting from the model optimization method. The FFV modeling and calculation process takes into account measurement errors at the both the experimental data and theoretical model levels. The key element is the accurate calculation of uncertainty through error propagation, which allows for a reliable interpretation of results. Molecular modeling and molecular dynamics programs are used for FFV modeling, including Materials Studio (BIOVIA), which allows for the calculation of the system volume and van der Waals volume based on the modeling of polymer structures. Materials Studio (BIOVIA) is a powerful tool for calculating system volume and van der Waals volume in polymer structures through advanced molecular simulation techniques. It integrates various methods, including molecular dynamics and Monte Carlo simulations, to provide accurate volumetric properties essential for understanding macromolecular behavior.

## 3. Results and Discussion

**Figure 2:**
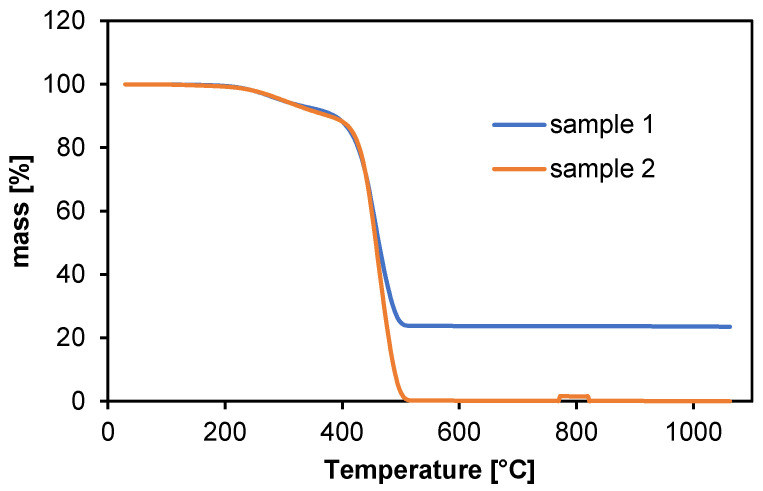
Percentage change in sample weight (thermogravimetric analysis).

During heating, changes in the mass of the samples as a function of temperature were recorded. The result of the analysis is a thermogram taking into account the chemical and physical changes related to heating, as shown in Figure 2.

In the low temperature range (30–200 °C), polyamide shows insignificant weight change, indicating good thermal stability and no significant degradation. During this phase, only minor evaporation of water or other volatile components may occur [18].

In the temperature range from 200 °C to 350 °C, a significant weight loss is observed, attributed to the degradation of thermal stabilizers and additives, such as plasticizers.

Significant changes in sample mass begin around 350–500 °C, where polyamide begins to lose mass faster. This is the primary degradation phase during which the polyamide polymer chains decompose. The sample mass decreases rapidly, which is characteristic of the thermal degradation of polyamides [19].

The highest degradation rate occurs at temperatures around 500 °C, which is typical for polyamides, often having a high degradation temperature related to their chemical stability [20,21,22,23,24,25,26,27,28,29,30,31,32].

It was observed that sample 1 contained a higher amount of thermally stable additives, as more than 20% of the sample mass remained after the temperature exceeded 500 °C. This indicates inorganic residues. In thermogravimetric analysis (TGA), the key supporting techniques are differential thermal analysis (DTA) and differential thermogravimetric analysis (DTG) [33].

Sample 1 may exhibit higher thermal stability compared to sample 2, potentially due to variations in polymer structure, additives, or processing conditions. This discrepancy could also arise from the presence of residual solvents or plasticizers in one of the samples, which may decompose at this temperature, leading to increased mass loss.

**Figure 3:**
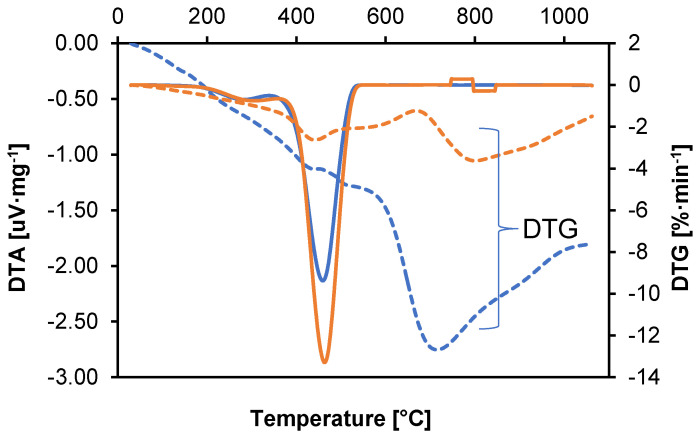
DTA and DTG as a function of temperature (blue line—sample 1, orange—sample 2).

Both of these techniques provide additional information that facilitates the understanding of thermal mechanisms and processes occurring in material samples. A graph showing the DTA and DTG values as a function of temperature is shown in Figure 3.

DTA analysis of samples shows troughs (endothermic peaks) that indicate heat-absorbing processes such as melting, evaporation, or decomposition. It determines the temperatures at which processes requiring heat absorption occur, e.g., evaporation of volatile components. An endothermic peak in the range of 30–150 °C indicates the evaporation of volatile components. The exothermic peak (around 400 °C) suggests the beginning of thermal degradation of polyamide [34]. The graph in Figure 3 shows DTG changes as a function of temperature for two polyamide samples. A material that shows stable thermal characteristics with minimal weight loss at elevated temperatures is likely to exhibit better resistance to hydrogen.

The peaks in the DTG plot indicate the temperatures at which the degradation rate is the highest. In the DTG plot, you can see separated peaks, which indicate a multi-stage degradation process. The height of the peaks indicates the intensity of the degradation processes. The highest DTG values were recorded in the initial phase of the process (30–150 °C), corresponding to the evaporation of volatile components. A significant difference in the speed of the process between samples 1 and 2 can be seen at temperatures above 600 °C, following the polyamide degradation phase, which indicates a significant difference in the additives contained in the polyamide. Higher decomposition temperatures suggest better thermal stability and resistance to hydrogen-induced degradation.

⮚ 50–150 °C.

Polyamides are hygroscopic, meaning that they absorb moisture from the environment. At this stage, water adsorbed on the surface and in the polymer structure evaporates.

⮚ 200–250 °C (depending on the type of polyamide).

Physical changes occur, such as relaxation of polymer chains or melting of polymer crystallites. A peak associated with the heat of fusion is visible on the DTA/DSC curve (differential thermal analysis or scanning calorimetry).

⮚ 250–350 °C.

In this phase, thermal degradation initiation processes occur:

  - Breakage of amide bonds (–CONH–).
  - Emission of volatile products, such as ammonia (NH_3_) and small amounts of CO_2_ and water.
  - These processes involve the decomposition of basic chemical bonds in the polyamide structure and the degradation of any chemical additives. In this respect, the thermal stability of the material is assessed; an important characteristic in the context of its use in high temperature conditions.
  - This difference suggests that exposure to hydrogen affects the thermal stability of the material, which may be related to decomposition or change in the stabilizing additives present.

⮚ 350–450 °C

In this phase, the main process of polyamide degradation occurs:

  - Polymer chains are depolymerized to monomers, such as caprolactam (for PA6) or diamine and hexanoic acid adipate (for PA66).
  - In an oxygen atmosphere, an oxidation reaction occurs, which leads to the formation of more CO, CO_2_, and other oxidation products.
  - A sharp mass drop is visible on the TG curve and an exothermic peak on the DTA curve (in an oxygen atmosphere).

⮚ 500 °C.

  - The first stage of decomposition involves the cleavage of amide bonds, leading to the formation of smaller organic molecules. Subsequent stages involve further decomposition of the organic residues to oxidation to simple compounds and gases. Residual analysis indicates the presence of organic residues, which may include stabilizing components and additives that may have been present or degraded to varying degrees by the exposure of sample 2 to hydrogen.

⮚ 650 °C

  - The main processes of polymer decomposition occur within this range. Intense drops in sample mass and rapid changes in DTG and DTA signals are noticeable, indicating the complete decomposition of the material into simpler compounds and gases. Further thermal changes are no longer significant for the structure of the material, as it has completely degraded. This phase of analysis provides complete information on the final degree of polyamide decomposition, which allows for a comparison of its thermal resistance with other materials and an assessment of its suitability in extreme conditions. TGA, DTA, and DTG analyses allows for a comprehensive assessment of the thermal stability of polyamide, revealing differences in the behavior of samples before and after exposure to hydrogen. This is crucial for assessing the barrier properties and strength of the material in applications where contact with hydrogen gas may affect its durability and functionality.

**Figure 4:**
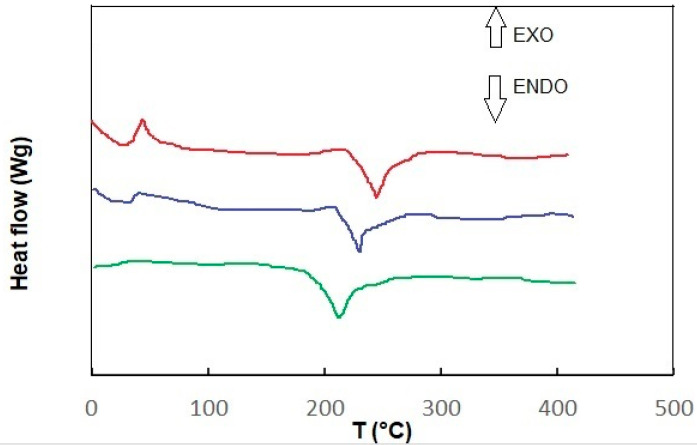
DSC of sample 1 (red line), sample 2 (blue line), and sample 2 after heating at 50 °C (green line).

Figure 4 shows the DSC measurement results for each sample, sample 1 and sample 2, at room temperature and after heating to 45 degrees. Depending on the type of sample, the glass transition temperature (glass transition initiation temperature T_ig_) and melting temperature (T_m_) vary according to the analyzed system.

As shown in the DSC profile of the tested sample, two peaks appear. The first one occurs at about 45 °C and the second one at 275 °C (sample 1). At approximately 45 °C, less heat is required to heat the tested sample (compared to the control sample). This may indicate that the tested sample is undergoing a crystallization process at this temperature, which is known to be an exothermic process [35]. The next transformation occurs at around 275 °C. Here, the heat flow rate to the tested sample (compared to the control sample) increases significantly, suggesting that endothermic processes are occurring in the tested sample. This may be a melting process, which is known to be endothermic. The DSC curve (thermogram) recorded for sample 2, which melted (endothermic process) in the tested temperature range during heating, showed a minimum shift of 230 °C, while during crystallization cooling, it showed 40 °C (exothermic process). The obtained thermogram allows one to determine the melting and crystallization temperature and the enthalpy of these transformations. In the case of heating, sample 2 (after contact with hydrogen) was heated before measurement, and the disappearance of the exothermic peak was observed (minimum trace around 35 °C) and a shift to 205 °C endothermic. As can be seen, recording differences in heat flow to the tested and control samples allows for precise calculation of the thermal effect of phase transitions [36].

Thermogravimetric differential thermal analysis (TG-DTA) and Differential Scanning Calorimetry (DSC) are essential techniques used to study the thermal properties of materials, particularly polymers like polyamide 66 (PA66) [37,38,39,40,41,42,43]. Comparing the results with other samples described in the publication, we can see in the analysis that a sample (approximately 5 mg of PA66) is placed in an aluminum pan and heated from room temperature to 600 °C at a rate of 10 °C/min under a nitrogen atmosphere. The TG curve indicates weight loss, which can be attributed to various factors, including the volatilization of water and low molecular weight components generated during polymerization. Significant thermal decomposition is observed between 350 °C and 450 °C, where rapid weight loss occurs, indicating the onset of degradation. The DTA curve shows an endothermic peak at 261 °C, which corresponds to the melting of crystalline PA66, while no significant thermal degradation is noted below 330 °C. Both TG-DTA and DSC are crucial for understanding the thermal behavior of materials like PA66. TG-DTA focuses on weight loss and thermal stability, while DSC provides insights into phase transitions. The combination of these techniques allows researchers to comprehensively analyze the thermal properties and degradation mechanisms of polymers, which is vital for applications in various industries.

In conclusion, TG-DTA and DSC are complementary techniques that provide valuable information about the thermal characteristics of materials, aiding in the development and application of polymers like PA66.

**Figure 5:**
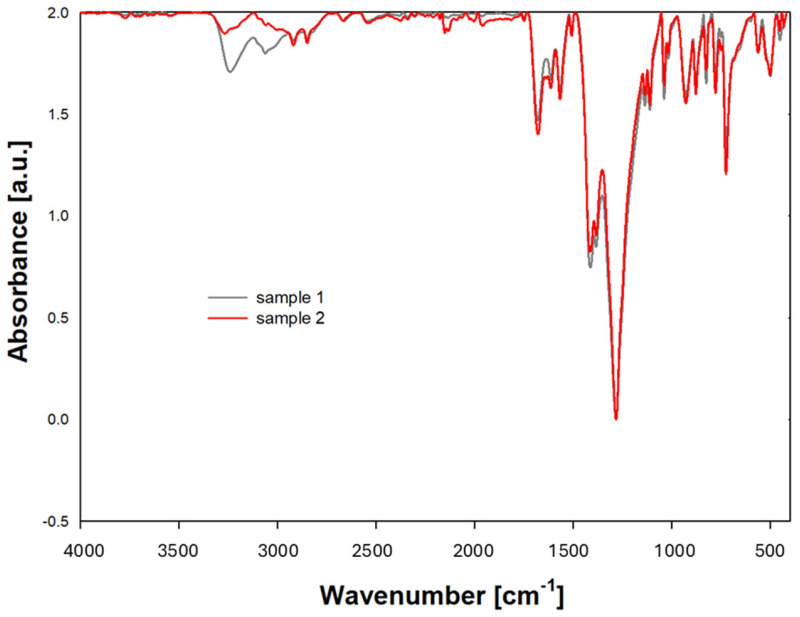
ATR analysis of the sample before hydrogen transfer (grey line) and of a flexible hose sample with a polyamide lining after hydrogen transfer (red line).

ATR spectra (Figure 5) were recorded on the absorbance scale in the wavenumber range from 4000 cm^−1^ to 350 cm^−1^. For each sample tested, 20 scans were performed to eliminate random interference. The analysis of the recorded oscillation-rotation spectra allowed conclusions about possible structural changes.

Identification of characteristic bands:

Typical absorption bands for amides (-CO-NH-) in polyamides occur in the ranges shown in Table 2.

Based on the presented ATR data, it can be concluded that the tested material contains characteristic polyamide functional groups, such as amide and methyl groups. A lower absorbance value was observed in the ATR analysis for the -NH and -C=O functional groups in the sample that was previously in direct contact with hydrogen. This means that the amount of IR (infrared) radiation absorbed by this functional group is lower compared to previous measurements. This may be due to several factors:

- Reduction in the concentration of -NH and -CO groups: if the sample has undergone chemical or physical degradation leading to the loss of -NH groups, then their concentration in the sample will be lower, resulting in a lower absorbance value.
- Chemical changes: if the -NH and -CO groups have undergone chemical changes (e.g., oxidation, reduction, and amidation), their characteristic absorption bands may change in intensity or shift, which may also lead to a decrease in the absorbance value. Hydrogen permeation in polyamide can potentially contribute to the loss of functional groups. Changes—their pace and degree—will depend on the conditions of use.

The functional groups in polyamides play a crucial role in determining their hydrogen permeation properties [44,45,46].

Amide groups (-CO-NH-): these groups are polar and can form hydrogen bonds, influencing the interaction between the polyamide matrix and hydrogen molecules. The degree of hydrogen bonding can either facilitate or hinder permeation depending on the network structure.

Hydroxyl groups can also form hydrogen bonds, which can trap hydrogen or create pathways for its movement, affecting overall permeation rates.

A decreased intensity of the band after hydrogen transport suggests that the sample has undergone a chemical modification or structural change that has impacted the activity of the IR-absorbing groups.

The presence of different functional groups can alter the flexibility of the polymer chains. Rigid structures tend to have lower permeability due to restricted movement, while more flexible structures can allow for easier diffusion of hydrogen.

Functional groups influence the crystallinity of polyamides. Higher crystallinity typically results in lower hydrogen permeability due to tightly packed molecular chains, while amorphous regions may provide additional pathways for gas diffusion.

The balance between hydrophilic (water-attracting) and hydrophobic (water-repelling) functional groups affects how hydrogen interacts with the polymer. More hydrophilic polyamides may allow for enhanced hydrogen solubility, thereby affecting permeation.

**Figure 6:**
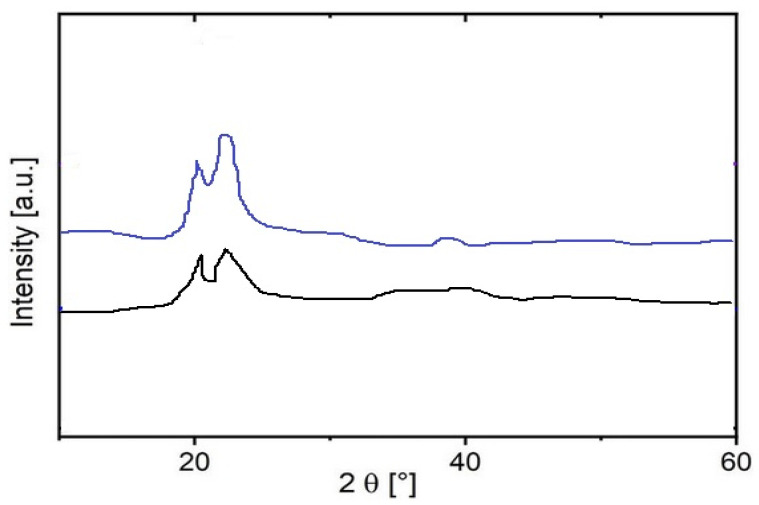
XRD of sample 1 (black line), sample 2 (blue line).

The crystal structures of sample 1 were analyzed using X-ray diffraction (XRD), and the effect of hydrogen interaction on these structures (sample 2) was also investigated. The XRD pattern (Figure 6) showed characteristic peaks at 2ϴ angles of 20.9° and 22.0° (Cu Kα), which are consistent with the typical profile of polyamide powder.

These peaks correspond to intermediate crystalline phases, α and γ, with calculated d-spacings of 0.425 nm and 0.404 nm, respectively [47,48]. Importantly, hydrogen permeation had no observable effect on the diffraction pattern (Figure 6), indicating that the crystalline structure of the polyamide remained intact despite exposure to hydrogen. This stability suggests that interaction with hydrogen does not significantly disrupt the ordered regions of the polymer, emphasizing the strength of the crystal skeleton in the polyamide.

**Figure 7:**
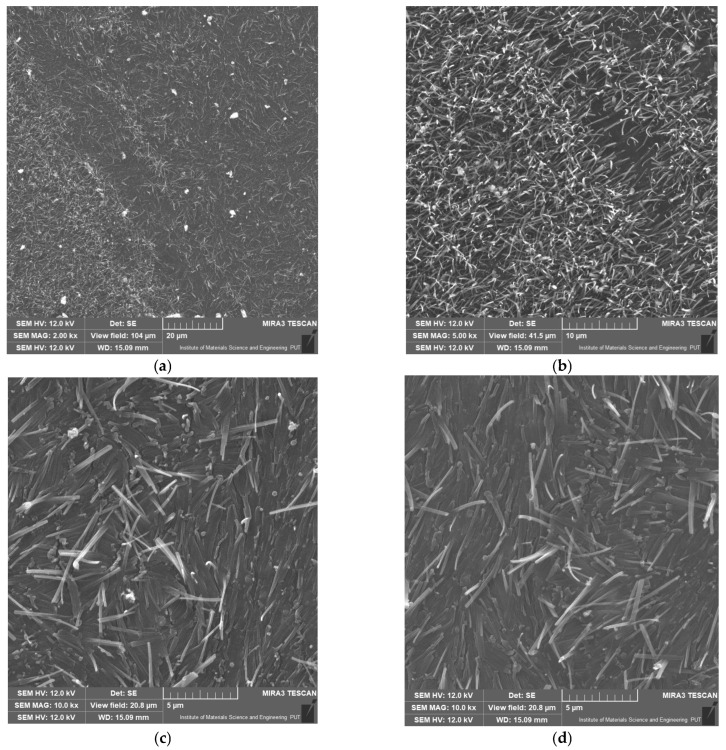
SEM images of the inner layer material of a polyamide (sample 1: (**a**–**c**); sample 2: (**d**)) hose at different magnifications.

SEM examinations of polyamide samples carried out at various magnifications provided detailed information on the microstructure of the material. Figure 7 shows the images of the sample at individual magnifications.

Most important observations:

- Small fibers were visible in all images, giving the surface a hairy-like appearance.
- The images revealed the granular structure of the material, especially visible at 15k× magnification, with visible, separated grains.
- No obvious defects, such as cracks or large defects, were observed, indicating good material quality.
- The structure of the polyamide was largely amorphous, without any distinct crystals, although the presence of ordered areas cannot be ruled out.
- In comparing SEM images for samples 1 and 2 at the same magnification of 5 um, it is evident that after hydrogen transfer, the morphology of the sample became smoother; however, no visible cracks were observed.

The SEM examination of polyamide (sample 1) allowed obtaining a detailed image of its microstructure, revealing key features such as roughness, granularity, and the lack of significant defects or air bubbles. These results are important in the context of testing hydrogen permeability through polyamide. The amorphizing structure of polyamide and the presence of fine pores may affect hydrogen diffusion, which is important for applications where controlling gas permeation is crucial. The high quality and homogeneity of the material, confirmed by SEM, may contribute to its effective use in applications requiring specific barrier properties.

Certain polymers exhibit varying effectiveness in reducing hydrogen permeation, which is crucial for applications involving hydrogen storage and transmission.

⮚ Polyamide (Nylon): is commonly used due to its good mechanical properties and moderate gas barrier capabilities. However, its effectiveness can vary based on the specific type of polyamide and any modifications made to enhance barrier properties.
⮚ Polyethylene (PE): high density polyethylene (HDPE) is often employed in hydrogen storage applications. While it has relatively high permeability compared to metals, its lightweight nature and cost-effectiveness make it a popular choice. Blending with other materials can improve its barrier properties.
⮚ Polyvinylidene Fluoride (PVdF): PVdF is known for its excellent chemical resistance and lower hydrogen permeability compared to other polymers. Its crystalline structure helps reduce gas diffusion rates, making it a suitable option for hydrogen applications.
⮚ Polysulfone (PSF): polysulfone exhibits good thermal stability and lower permeability to gases, including hydrogen. Its structural integrity at elevated temperatures makes it a viable candidate for high performance applications.
⮚ Polycarbonate (PC): polycarbonate has been shown to have lower hydrogen permeability than many other polymers, making it useful in applications where reduced gas diffusion is critical.
⮚ Metal-organic frameworks (MOFs) and polymer composites: incorporating MOFs within polymer matrices can significantly enhance barrier properties against hydrogen permeation. These composites leverage the high surface area and selective adsorption characteristics of MOFs to reduce overall gas permeability.

The choice of polymer for hydrogen storage applications should consider not only the inherent gas barrier properties but also factors such as mechanical strength, thermal stability, and compatibility with other materials [49,50,51,52,53,54].

**Figure 8:**
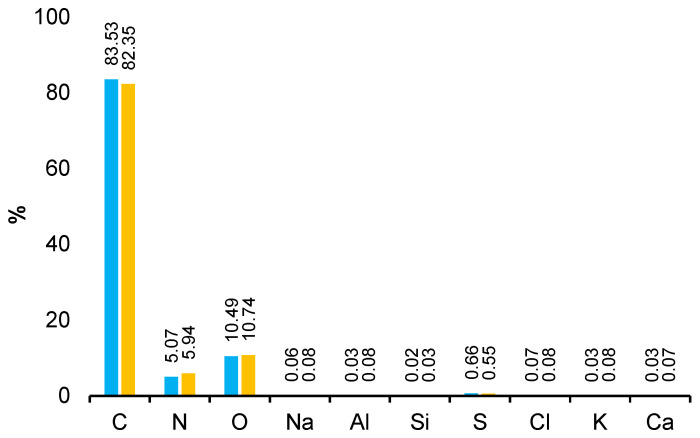
Percentage content of elements in the material based on EDS analysis, 20 kV (blue) and 12 kV (orange) of sample 1.

The obtained EDS spectra allowed for the identification of characteristic peaks corresponding to individual elements, including carbon (C), nitrogen (N), and oxygen (O). The analysis was carried out at two voltage values: 12 kV and 20 kV, which allowed for the assessment of the internal structure of the material at different depths of radiation penetration. The increase in voltage increased the intensity of the signal of elements with a higher atomic mass, enabling a more accurate determination of their distribution and quantity. The percentage content of the elements is presented in the graph (Figure 8). The high carbon content in the polyamide favors the formation of a compact, dense structure that limits hydrogen permeability. In carbon-based polymers, increasing the carbon ratio in the backbone chains often leads to improved π-π stacking and crystalline domains, particularly in condensed systems. The energetics of phase formation favor crystalline structures over amorphous ones when the carbon concentration exceeds a critical threshold. Sufficient carbon content can influence the nucleation and growth of crystalline domains during cooling or annealing, leading to higher degrees of crystallinity.

Carbon, nitrogen, and oxygen, occurring in amide and carbonyl groups, create a network of hydrogen bonds and interactions that limit hydrogen penetration through the material. Components such as C, H, and N are key in the context of interaction with hydrogen: carbon contributes to the formation of the crystalline phase, while nitrogen and oxygen, as part of amide groups, can form hydrogen bonds, reducing hydrogen permeation.

At 12 kV, light elements produce stronger signals due to reduced background noise, whereas at 20 kV their signal strength may decrease as the beam penetrates deeper into the sample, diluting surface-specific contributions. At 12 kV, heavy element signals may be weak or absent if the beam energy is insufficient to overcome excitation thresholds. At 20 kV, heavy elements exhibit much stronger signals due to their appropriate excitation energy. Combining data from 12 kV and 20 kV enables efficient detection of both light and heavy elements. 12 kV highlights surface features, while 20 kV provides information on bulk composition. The dual-voltage approach improves the accuracy of element quantification by separately addressing surface and bulk contributions.

The levels of carbon, nitrogen, and oxygen in polyamides have a key influence on hydrogen permeability, mainly through chemical and morphological interactions. EDS results, combined with SEM and techniques such as FTIR or XRD, provide a complete picture of the relationship between the material structure and its transport properties.

**Figure 9:**
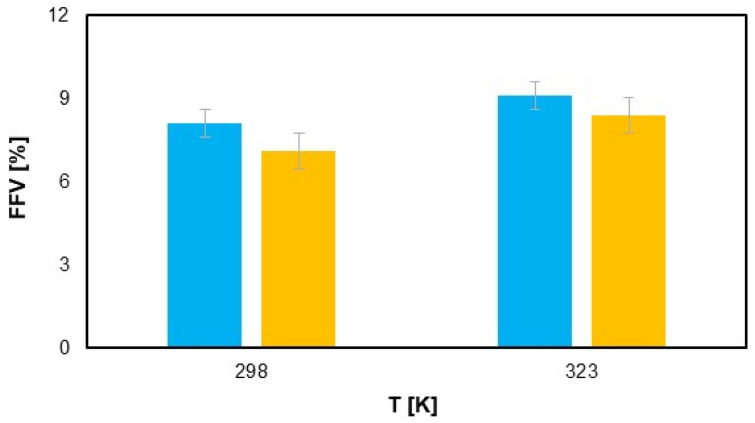
Plot of FFV content in sample 1 (blue) and 2 (yellow) at various temperatures.

Fractional free volume (FFV) is a crucial concept in the study of materials, especially polymers, as it relates to the unoccupied space within a material’s structure. This free volume plays a significant role in determining various physical and chemical properties, including diffusion, mechanical behavior, and thermal stability. The size of the FFV and the distribution of free volume within the unit cell will influence the ability of gas molecules to diffuse within the material. By examining the free volume of polymers, the gas barrier properties of materials can be analyzed and explained under a microscope. The fractional free volume can be calculated according to the following formula [55]:

(1) $$FFV=\frac{V_{F}}{V_{F}+V_{0}}\cdot 100\%$$

where *V_F_* refers to the “free volume”, the free volume, and *V*_0_ refers to the “occupied volume” of the polymer. The FFV at 25 °C and 50 °C (experimental part) models were calculated after optimization. According to the data [32], the radius of the hydrogen atom is 1.20 A, and the data on the distribution of the free volume, free volume, and occupied volume of polyethylene and polyamide at different temperatures were obtained by simulation. Figure 9 illustrates that the FFV of the polyamide samples increases with increasing temperature, which shows the thermal expansion of the polymer matrix. This trend is noticeable for both the first and second samples. However, under the same temperature conditions, the samples exposed to hydrogen show a slight reduction in FFV. Although this difference is minimal—0.3% at room temperature and 0.5% at elevated temperatures—it indicates that hydrogen permeation can cause subtle changes in the polymer structure, probably due to interactions at the molecular level. Nevertheless, these changes remain in a range that does not significantly affect the overall performance of the material, confirming that hydrogen permeation did not have a major impact on the FFV [56]. This observation suggests that although hydrogen exposure may slightly affect the free volume, the structural integrity of the polyamide is largely preserved, making it a suitable material for applications involving hydrogen transport under typical operating conditions.

The labeled error measurement on the graph illustrates the uncertainty due to the simulation deviations for the FFV [%] value as a function of temperature (T [K]). The error bars show the range of deviations for each temperature, considering the repeatability and accuracy of the simulation, indicating that the data are reliable within the specified errors.

### 3.1. Discussion of the Phenomenon of Cross-Fertilization

Polymeric materials are often used for their good barrier properties in many important practical applications. The penetration of gases through polymeric materials is an important phenomenon in various areas, such as medicine or the automotive industry. Polyamide (PA) and polyethylene (PE) are among the most commonly used polymers, differing significantly in their gas barrier properties. Understanding the mechanism of gas permeation through these materials requires analysis of the physical laws and chemical structures of these polymers. The transport of gas through a polymer membrane can be defined as the property of this material to penetrate to allow gas molecules to penetrate and pass through. A polymer membrane is considered a homogeneous, non-porous material at a given temperature. This process described by the mechanism of solution diffusion [57,58].

- Diffusion through the boundary layer of the side corresponding to the higher partial pressure (upstream side);
- Gas absorption (by chemical affinity or solubility) by the polymer. Gases adsorb on the polymer surface according to the Henry’s equation:
  (2) $$C=k_{H}P$$
  where C is the gas concentration in the polymer, $k_{H}$ is Henry’s coefficient, and P is gas pressure.
- Gas diffusion occurs inside the polymer membrane, following Fick’s first law:
  (3) $$J=-D\frac{dC}{dx}$$
  where J is the diffusion flux, D is the diffusion coefficient, and $\frac{dC}{dx}$ is the concentration gradient.
- Desorption of the gas on the lower partial pressure side;
- Diffusion by the boundary layer on the lower side [57,58].

**Figure 10:**
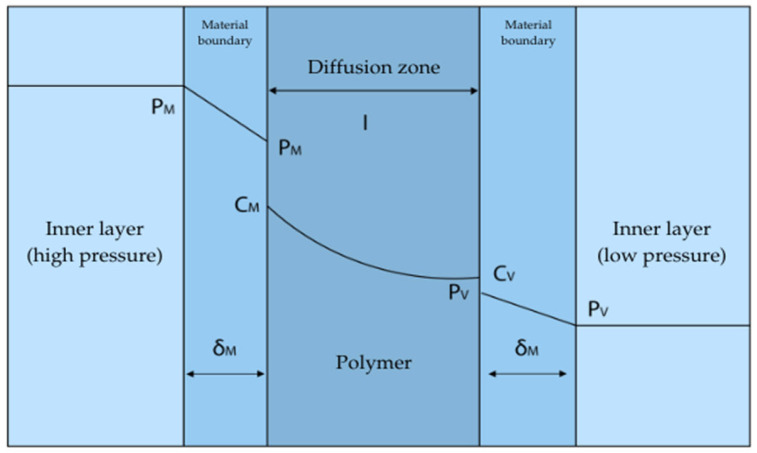
Scheme of substance permeation through the polymer [59].

The diagram of the medium’s penetration through the polymer material is shown in Figure 10.

The volume of hydrogen released by the material will depend on the material’s permeability coefficient, internal surface area, material thickness, contact time, and gas pressure:

(4) $$V_{H_{2}}=\frac{P_{H_{2}}\cdot A\cdot t\cdot p}{l}$$

where: $V_{H_{2}}$—Volume of hydrogen diffusing through the sealing insert, cm^3^; $P_{H_{2}}$—Hydrogen permeability coefficient, cm^3^ [STP]·cm·cm^−2^·s^−1^·cmHg^−1^ (constant value for the material); A—Tank area, cm^2^; t—time, s; p—gas pressure, cmHg (1 bar = 75 cmHg); l—Insole thickness, cm [60].

The rate of hydrogen penetration through the polymer lining is influenced by the type of material used, pressure, and operating temperature [61]. Compared with polyethylene, polyamide has better resistance to hydrogen penetration, which indicates that polyamide is a good material for the inner layer of type IV tanks. Polyamides have a crystalline structure, which increases their gas barrier properties. Strong hydrogen bonds between amide groups additionally impede gas diffusion. The gas diffusion coefficient of polyamide is relatively low, which means that this material is a good barrier to gases such as hydrogen. High density polyethylene (HDPE) also has some gas barrier properties, although not as high as polyamide. The gas diffusion coefficient of polyethylene is higher than that of polyamide, making it a less effective gas barrier. Comparison of the barrier properties of polyamide and polyethylene:

Diffusion coefficient (D)

(5) $$D_{PA}<D_{PE}$$

Solubility coefficient (S)

(6) $$S_{PA}\approx S_{PE}$$

Transmission coefficient (P)

(7) $$P_{PA}<P_{PE}$$

Polyamide has better gas barrier properties than polyethylene, mainly due to its lower diffusion coefficient. The crystalline structure and hydrogen bonds in polyamide make it much more difficult for gases to pass through, while the more amorphous structure of polyethylene allows for easier diffusion [61].

### 3.2. Solubility Coefficient (S)

The solubility coefficient (S) reflects the thermodynamic characteristics of the interaction between polymeric materials and gas-permeable molecules. This is a thermodynamic parameter.

**Figure 11:**
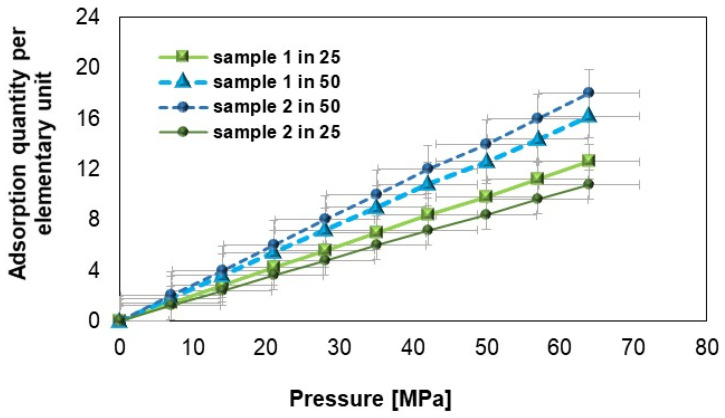
Adsorption isotherms (at constant temperatures) as a function of pressure for samples 1 and 2.

Figure 11 shows that under identical conditions of pressure and temperature, the number of hydrogen molecules adsorbed by samples 1 and 2 (after hydrogen flow) differs. As the pressure increases, an increase in the number of hydrogen molecules adsorbed is observed, while increasing the temperature leads to a decrease in the number of molecules adsorbed. For the same pressure and temperature parameters, the mass of adsorbed molecules in sample 1 is always greater than in sample 2 [61]. The increase in pressure causes a gradual increase in the amount of adsorbed substance, and the differences between the samples and proportions indicate their specific adsorption properties. The marked error squares on the graph represent the uncertainty range for each measurement point in relation to the amount of adsorption as a function of pressure. Their presence emphasizes data reliability (error squares show the limits of deviations that may result from instrumental uncertainty, sample variability, or measurement conditions); sample comparison (based on the size of the errors, it is possible to assess which samples are more stable and have smaller deviations in the tested pressure range); and measurement stability (an even distribution of errors suggests that the measurements are systematic, while large differences between error values at individual points may indicate specific effects, e.g., related to adsorption dynamics). The error bars show that despite the approximate nature of the data, the variability of the results is within an acceptable range, confirming their consistency and reliability within the error limits.

**Figure 12:**
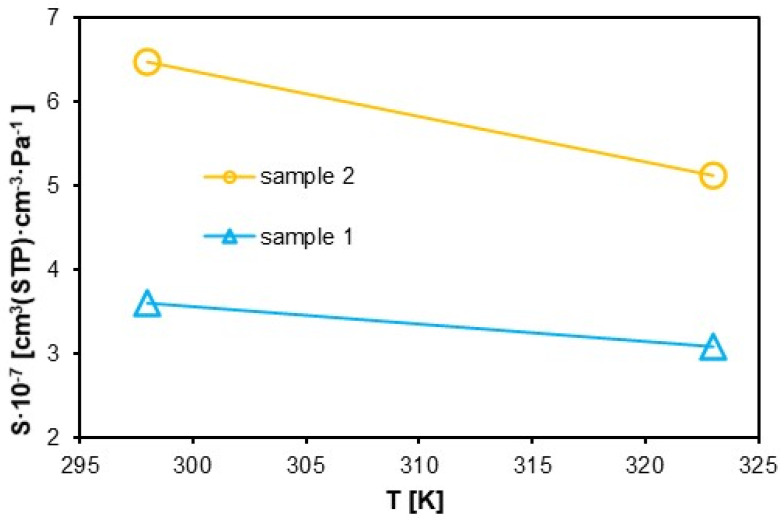
Solubility coefficient of sample 1 and sample 2 at 20 MPa.

**Figure 13:**
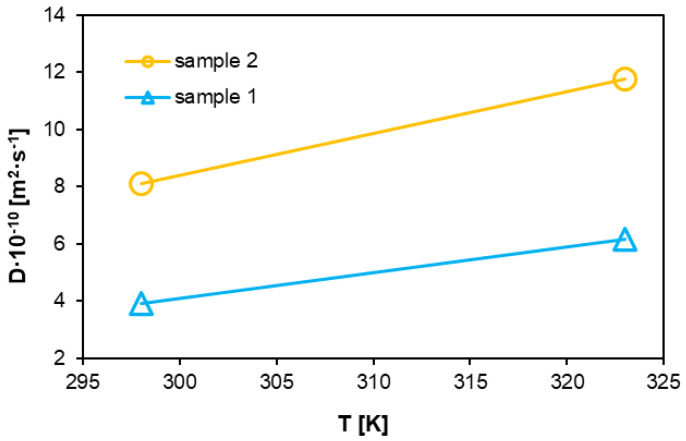
Diffusion coefficients of samples 1 and 2 at 20 MPa.

The graph in Figure 12, on the other hand, was developed using the least-squares method, fitting the adsorption isotherms. The solubility of the polymers decreases with increasing temperature, with the solubility coefficient for sample 1 being significantly lower than for sample 2. For sample 1, the solubility coefficient decreases with increasing temperature from a value of approximately 3.5·10^−7^ to 3·10^−7^ cm^3^·cm^−3^·Pa^−1^, and sample 2 from 6.5·10^−7^ to 5·10^−7^ cm^3^·cm^−3^·Pa^−1^. The trends of the solubility (Figure 12) and diffusion (Figure 13) coefficient curves obtained is consistent with the results in [56,62], but the results remain slightly different.

### 3.3. Diffusion Coefficient

The diffusion coefficient represents the mass or number of moles of a substance diffusing vertically through a unit area under a unit concentration gradient per unit time in the direction of diffusion. Various factors influence the diffusion coefficient, but it is ultimately directly related to the free volume [63]. Under high pressure, polymer molecular chains arrange themselves more tightly and in a more ordered manner, reducing the polymer’s free volume and making it harder for hydrogen molecules to diffuse.

Conversely, as the temperature increases, the thermal motion of polymer molecular chains intensifies, resulting in greater relative displacement between the chains. This leads to an increase in free volume and, consequently, in the diffusion coefficient [64]. Higher temperatures also enhance the kinetic energy of hydrogen molecules, making them more prone to diffusion. These volume changes are illustrated in Figure 12. The graph in Figure 13 shows changes in both analyzed systems, with identical trends observed. The second sample, representing the system exposed to hydrogen for 10 h, exhibits higher diffusion coefficient values. The change in the coefficient as a function of increasing temperature follows a similarly linear trend as that of the first sample. However, the values for system 2 are nearly twice as large as system 1. Additionally, as the degree of polymerization increases, the molecular chain length within the polymer grows. This weakens the mobility of chain segments, making it more challenging to form diffusion pathways for gas molecules, thereby hindering their diffusion and reducing the diffusion coefficient.

Hydrogen as a gas with a small molecular diameter (0.29 nm) has the ability to penetrate polymeric materials, but this process is limited by the local density of the chains and their dynamics. After exposure to hydrogen (sample 2), it can be observed that hydrogen induced slight changes in the microporosity of the structure, which, in turn influenced local changes in the diffusion coefficient.

An increase in temperature is a factor that reduces the order of molecular chains by increasing thermal energy. In sample 2, an increase in temperature could have promoted hydrogen diffusion while increasing the free volume and kinetic energy of the molecules.

## 4. Conclusions

To improve polyamide materials for hydrogen transport, efforts should focus on a few aspects: reducing hydrogen permeability through nanoparticle fillers and nanocomposite creation; enhancing mechanical and chemical resistance via copolymerization with durable materials and fiber reinforcement; increasing thermal stability through stabilizing additives and molecular modifications; and minimizing water absorption using hydrophobic modifications and cross-linking. The most significant changes in the material’s structure occur within the temperature range of 200–350 °C, which is critical when searching for a material resistant to the effects of transported hydrogen. According to DTA and DTG analyses, a higher decomposition temperature indicates better resistance to the degrading effects of hydrogen. The analyzed material exhibits a crystalline structure typical of polyamides, which influences its interactions with hydrogen. Simultaneously, no substantial structural changes are observed, confirming its suitability for hydrogen transport. SEM image analysis reveals satisfactory resistance to the degrading effects of hydrogen in the polyamide-containing material. The presence of the amide group limits hydrogen permeation through the material by forming hydrogen bonds.

The analysis of the solubility coefficient (S) and diffusion coefficient (D) revealed significant differences between sample 1 and sample 2. The solubility coefficient for Sample 1 (3.8·10^−7^ cm^3^·cm^−3^·Pa^−1^ at 300 K) is significantly lower than that of sample 2 (6.5·10^−7^ cm^3^·cm^−3^·Pa^−1^ at 300 K). This difference can be attributed to variations in the material’s structure and microporosity, especially after hydrogen exposure in sample 2. These changes influence the material’s ability to adsorb and dissolve hydrogen molecules.

As the temperature increases, the material’s solubility decreases, which is consistent with literature findings. At 320 K, the solubility coefficient drops to 3.2·10^−7^ cm^3^·cm^−3^·Pa^−1^ for sample 1 and 5.5·10^−7^ cm^3^·cm^−3^·Pa^−1^ for sample 2. This decrease may be due to the expansion of the polymer network and the reduction in the number of active sites available for hydrogen adsorption. In sample 2, after hydrogen exposure, changes in microporosity occur, facilitating hydrogen diffusion by increasing the free volume and the kinetic energy of the molecules. In contrast, sample 1 maintains a more ordered structure, making the diffusion process more difficult.

The diffusion coefficients indicate that at high pressure and temperature, hydrogen diffusion in both materials is limited by the density and dynamics of polymer chains. The diffusion coefficient values increase with temperature—for sample 1, they are 4.2·10^−10^ m^2^·s^−1^ (300 K) and 6.5·10^−10^ m^2^·s^−1^ (320 K), whereas for sample 2, they are 8.5·10^−10^ m^2^·s^−1^ and 12.5·10^−10^ m^2^·s^−1^, respectively. The higher values for sample 2 suggest that this material allows greater freedom of movement for hydrogen molecules, likely due to a more developed microporous structure.

Furthermore, hydrogen adsorption analysis indicates that the amount of adsorbed gas increases with pressure. For example, at 50 MPa, the amount of adsorbed hydrogen for sample 1 at 25 °C is approximately 10 units, while at 50 °C, it rises to 14 units. For sample 2, these values are 12 and 17 units, respectively, suggesting that sample 2 has a greater capacity for hydrogen adsorption under the tested conditions.

These differences may result from the presence of a higher number of active sites in sample 2, which can bind hydrogen more effectively, even under elevated pressure conditions. This suggests that sample 2 could be a better candidate for applications requiring efficient hydrogen storage, such as hydrogen-based technologies used in the automotive and energy industries.

Further research on microporosity and material structure, for example, using spectroscopic or microscopic techniques, could provide more detailed insights into the mechanism of hydrogen transport in the studied samples. Another important aspect to consider is the long-term stability of these materials under cyclic pressure and temperature changes, which could help optimize their practical industrial applications. Improving adhesion in composites by functionalizing nanofillers and using plasticizers. Each of these modifications can significantly enhance the properties of polyamides, making them more effective in applications related to hydrogen transport.

## Figures and Tables

**Table 1:** Advantages and limitations.

Feature	Polymer Liners	Metal Liners
Hydrogen permeability	Higher permeability; potential for significant losses	Lower permeability; minimal losses
Weight	Lightweight and flexible	Heavier; less flexible
Structural integrity	May require reinforcement for high pressure	Excellent under high pressure
Cost	Generally lower cost	Higher manufacturing costs
Corrosion resistance	May degrade over time	Can corrode; requires protective measures

**Table 2:** Results obtained from ATR.

Wavenumber (cm^−1^)	Band
3300–3500	N-H stretching
1640–1690	C=O stretching (carbonyl group)
1540–1560	C-N stretching and N-H deformation (so-called amide II band)

## Data Availability

Data presented in this study are available on request from the corresponding author due to: raw data will be made available on specific requests.

## Supplementary Materials

The following supporting information can be downloaded at: https://www.mdpi.com/article/10.3390/ma18071402/s1, Table S1: Comparison of the physical properties of selected polymers; Table S2: Functional groups in polymeric materials and their influence on gas permeation.
