# Supplementary material for: Analysis of the Strength of Polyamide Used for High Pressure Transmission of Hydrogen on the Example of Reinforced Plastic Hoses
Source: Materials (Basel). 2025 Mar 21;18(7):1402. doi: 10.3390/ma18071402 (PMC11989395; doi:10.3390/ma18071402)
Supplement: Supplementary file 1 [file materials-18-01402-s001.zip › materials-3488172-supplementary.pdf]

Analysis of the strength of polyamide used for high-pressure transmission of hydrogen on the example of reinforced plastic hoses

Natalia Dawicka [1], Beata Kurc [2,*], Xymena Gross [3], Jakub Tomasz [3], Katarzyna Siwińska-Ciesielczyk [4] and Agnieszka [1] Poznan University of Technology; n.dawicka4@gmail.com;

[2] Institute of Chemistry and Electrochemistry, Faculty of Chemical Technology, Poznan University of Technology, Berdychowo 4, PL-60965 Poznan, Poland; beata.kurc@put.poznan.pl

[3] Poznan University of Technology, Poland, Student; xymena.gorka@student.put.poznan.pl, jakub.tomasz@student.put.poznan.pl

[4] Institute of Chemical Technology and Engineering, Faculty of Chemical Technology, Poznan University of Technology, Berdychowo 4, PL-60965 Poznan, Poland, katarzyna.siwinska-ciesielczyk@put.poznan.pl, agnieszka.kolodziejczak-radzimska@put.poznan.pl

* Correspondence: beata.kurc@put.poznan.pl,

Table S1. Comparison of the physical properties of selected polymers

	**PTFE**	**PA6**	**PA11**	**PA12**	**HDPE**	**SBR**	**LDPE**
min T [°C]	-268	-70	-54	-60	-60	-85	-20
max T [°C]	260	82-104	82-104	80-104	80-100	70-75	150
permeation 25°C mm³/m² 24hr-atm							
N_2	193	0,3	1,2	6	18	0.2	1.5
O_2	390	1,2	13	20-36	44-73	15	54
**H_2**	**866**	**35-43**	**127**	**127**	**126**	**150**	**300**
CO_2	1100	2	60-140	60-140	136-228	-	45

Most Type IV hydrogen storage tanks are lined with polyethylene [15] as the main material. However, polyamide and modified polyamide [16] are also considered as lining materials due to their good mechanical and barrier properties. Toyota Motor Corporation uses hydrogen tanks lined with polyamide in the Toyota Mirai hydrogen vehicle. The possibility of using polyethylene and polyamide in hydrogen storage and transport applications was investigated by Fang-Qing, Ji Dong-Mei [5]. The process of diffusion and adsorption of hydrogen particles in these materials was examined and the influence of the solubility coefficient, diffusion coefficient and free volume distribution (FFV coefficient) on the permeability of gas particles was analyzed. The mechanism of hydrogen permeation in polymeric materials was investigated on a microscopic scale.

❖ *The influence of functional groups on gas permeation*

Table S2. Functional groups in polymeric materials and their influence on gas permeation [33-39].

Functional group	Chemical structure	Impact on gas permeation
Hydroxyl	R-OH	Creates hydrogen bonds, increases density, reduces free volume
Carbonyl	R-C=O	Creates hydrogen bonds and increases the polarity of the material
Carboxyl	R-COOH	Creates hydrogen bonds and ionic interactions, seals the polymer matrix
Amine	$R\text{-}NH_2$	Creates hydrogen bonds and enhances barrier properties
Nitrile (-CN)	R-CN	Increases polarity, stronger intermolecular forces, reduced permeability
Chloro (-Cl)	R-Cl	Increases density and crystallinity, reduces permeability
Aryl	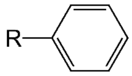	Increases stiffness and density, reduces free volume
Sulfonyl	$R\text{-}SO_3H$	Increases the ionic character and strengthens intermolecular interactions

Hydrogen gas, due to its small particle size and high diffusivity, can easily penetrate polymeric materials. The materials used to transmit hydrogen require a specific structure, with minimal free volume and strong inter-molecular bonds. The functional groups discussed above can be strategically incorporated into polymer chains to improve their hydrogen barrier properties. Incorporation of polar groups: such as hydroxyl, carboxyl and nitrile groups, enhance intermolecular interactions, reducing the free volume and creating a tighter polymer matrix that is less permeable to hydrogen [33, 34, 36]. Functional groups such as chlorine increase the crystallinity of the polymer, creating a more ordered structure that hinders hydrogen diffusion [37]. Aromatic rings and bulky substituents can increase the density of the polymer, limiting the routes available for hydrogen permeation [38, 39]. Functional groups capable of forming hydrogen bonds, such as hydroxyl, amino and carboxyl groups, create strong intermolecular networks that further reduce permeability [33-36].

❖ *Consequences of low barrier properties*

The low barrier properties of polymeric materials have important consequences, especially in the context of hydrogen storage and transport. Hydrogen is the smallest and lightest of the gases, making it exceptionally difficult to be retained by barrier materials.

- the low barrier properties of polymers lead to potential hydrogen leakage, which is a serious safety hazard, especially in closed spaces. Hydrogen is flammable and can lead to explosions under the right conditions.
- hydrogen is a valuable fuel, and its loss during storage and transport reduces the efficiency of hydrogen-based energy systems.

- hydrogen penetration through polymers may lead to their degradation, especially with long-term exposure to hydrogen. Material degradation can reduce its mechanical strength and barrier properties, which can lead to failure over time. Thomas et al. [15] examined the behavior of the polyethylene inner layer of a type IV hydrogen storage tank.
- hydrogen can influence the mechanical properties of polymers. It may lead to swelling of the material, reducing its strength and elasticity. Assuming there are many tiny pores inside the plastic, as hydrogen diffuses, hydrogen will fill the holes and the pressure will always increase. When the pressure in the holes is greater than the yield strength of the material, a bubble will form.

❖ **More information about directions of further research**

High-temperature rearrangement and carbonization enable it to achieve UL-94 V-0 test results, reducing peak heat release by 41% and total heat release by 50%. Hydrogen bonding improves polymer chain symmetry, improving mechanical properties and barrier performance. Low dielectric properties and high thermal stability make it suitable for advanced polymer applications [40-42]. Inspired by nylon and Kevlar, the new polyamide with sulfide and reversible hydrogen bonds achieves a tensile strength of 1180 MPa and an energy absorption of 433 MJ/m^3, surpassing Kevlar. This material is an environmentally friendly alternative to synthetic fibers and a promising barrier to hydrogen transfer [43, 44]. Furthermore, biochar from pyrolyzed biomass was incorporated into PA11 (polyamide 11) by in situ polymerization, which improved mechanical and thermal properties. SEM analysis confirmed excellent interfacial compatibility. The composite with up to 50 wt.% biochar increased the tensile strength by 35% and the modulus by 72%, making it ideal for hydrogen transport applications. Furthermore, 3D printing of biochar-PA11 fibers showed high dimensional accuracy and defect-free results. This material supports fluorine-free coatings, reducing environmental impact and minimizing waste. The development of these sustainable, high-performance materials solves key engineering challenges while promoting safer, more durable alternatives.

❖ *1.3.1. Challenges in hydrogen permeation through polymers*

One of the most significant challenges associated with the use of polymer materials, such as polyamide, in hydrogen storage and transport is the issue of gas permeation. Hydrogen, being the smallest molecule, has a high diffusion rate through many materials, including polymers. This can result in hydrogen loss over time, reduced efficiency, and safety concerns. The permeation of hydrogen through polymer liners is influenced by several factors, including:

- **Material composition**: different polymers have varying degrees of permeability. For example, polyamides generally offer better resistance to hydrogen permeation compared to other thermoplastics, but they still require modification to meet industrial standards for high-pressure hydrogen applications.
- **Temperature**: higher temperatures increase the kinetic energy of hydrogen molecules, leading to faster diffusion through the polymer matrix. This is particularly important in applications where the system undergoes large temperature fluctuations, such as during the compression and decompression of hydrogen .
- **Pressure**: increase raises the concentration of hydrogen molecules in contact with the polymer, thereby increasing the permeation rate. This is a key concern in high-pressure applications, such as in hydrogen storage tanks or transmission hoses.

❖ *1.3.2. Modifications of polyamids for improved hydrogen barrier properties*

To address the challenges posed by hydrogen permeation, research has focused on modifying polyamides and other polymers used in hydrogen storage systems. Some of the key approaches include:

- incorporating nanofillers, such as graphene or montmorillonite, into polyamide matrices can significantly improve gas barrier properties. These nanocomposites create tortuous diffusion pathways for hydrogen molecules, reducing the overall permeation rate .
- applying thin layers, such as silicon oxide or aluminum oxide, onto the polymer surface can act as an additional protective layer, preventing hydrogen from penetrating the material.
- introducing crosslinking agents into polyamide structures increases the density and rigidity of the polymer matrix, making it more resistant to hydrogen diffusion. Chemical modification of polyamide chains, such as through copolymerization with low-permeability materials, can also enhance gas barrier properties .

The aim of this study is to investigate the effects of hydrogen exposure on the properties of polyamide, a material that plays a critical role in hydrogen storage applications. The analysis was conducted on two samples: one before and the other after 10 hours of hydrogen flow exposure, allowing for an assessment of structural and property changes under these conditions.

Although this work focuses on a single material and a limited number of samples, it represents an important preliminary step toward understanding the mechanisms of polymer material degradation caused by hydrogen exposure. The issue of material degradation in such applications is significant, and the number of available scientific reports in this field is limited. Therefore, the results of this analysis can serve as a valuable foundation for further research, including comparative studies of different materials, such as other polymers and composites, to identify optimal material solutions for hydrogen storage technologies.
